# The Shortest Follies Are the Best

**DOI:** 10.3201/eid1803.AC-1803

**Published:** 2012-03

**Authors:** Polyxeni Potter

**Affiliations:** Centers for Disease Control and Prevention, Atlanta, Georgia, USA

**Keywords:** art science connection, emerging infectious diseases, art and medicine, Paulus Potter, God Appearing to Abraham at Sichem, the shortest follies are the best, animals, cows, livestock, bovine tuberculosis, about the cover

“There is a kind of commerce betwixt beasts and us, a certain relation and mutual obligation, whereof there is no other reason, but that they belong to one and the same master, and are of the same family that we are,” wrote French philosopher and theologian Pierre Charron. Not a new idea, this partnership between humans and animals dates back 9,000 years to the domestication of animals in the Stone Age. And as far back as the Paleolithic Age, art on cave walls suggested a close, if perilous, human association with wild and dangerous beasts—a short folly, not common today, except among hunters and adventurers.

Animals feature prominently in art of all ages, and in the 17th century, no longer as part of human scenes but as the primary subject matter. This shift signaled a change in the balance of power between owner and subservient beast, between humans and work animals. Dutch painter Paulus Potter understood this and expressed it by moving away from the human figure, traditionally the artist’s “noblest calling.” Some of his paintings contained no humans at all, bringing instead to center stage the horse or milk cow, the donkey or the mule, which until the advent of machines, were primary providers of agricultural, industrial, and other services.

Potter received early instruction from his father, a noteworthy landscape and figure painter, and from Nicolaes Moeyaert and others, who painted biblical and mythologic scenes. But soon he took off in his own direction, precocious, prolific, and popular, learning from nature and painting furiously, as if anticipating his demise before the age of 30 from tuberculosis. He joined the guild of St. Luke in Delft, married well, and was soon noticed by Dutch surgeon and mayor of Amsterdam Nicolaes Tulp, who had also earlier commissioned young Rembrandt van Rijn to paint the famed *Anatomy Lesson of Dr. Nicolaes Tulp*. At the mayor’s invitation, Potter moved to Amsterdam, where, like Rembrandt before him, he became Tulp’s protégé. The portrait of Tulp’s equestrian son Dirk was Potter’s last work. His painting *The Young Bull* (1649) was to become as famous as Rembrandt’s *Night Watch.*

During his brief artistic career, Potter became the most famous animalier of his day and a pioneer in featuring farm beasts outside the biblical or mythologic context. His approach influenced the presence and appearance of animals in European art. He painted them with sensitivity and exquisite naturalism in all their activities: standing, eating, drinking, sleeping, at work or at rest; integrated them in local valleys, trees, and waterfalls; and showed them in perfect harmony with nature, as humans rarely are.

The appearance and behavior of the diverse species of animals painted by Potter illustrate their interaction with humans during the 17th century, a period of land reclamation and sweeping socioeconomic and religious changes in Holland. All over Europe, agricultural production was increasing as a result of new technologies, ushering in unprecedented prosperity. An expanding art market brought in new buyers, whose perceptions and interpretations of the role and image of animals in society influenced artistic styles. At this time in Holland, the well-groomed milk cow was a symbol of stability and peace.

*God Appearing to Abraham at Sichem* is a bucolic scene with a biblical bent. A posthumous portrait of Potter by Bartholomeus van der Helst closely resembles the face of Lot in this painting—a self-portrait of Potter at age 16. The artist’s father may be depicted as Abraham; his mother as Sara. The patriarchal family is anchored against a tree in the corner of the canvas. In the center, two prominent milk cows gaze directly at the viewer. The carefully constructed landscape is traversed by a convoy of animals and their attendants as far as the eye can see. This is a fine spot with a sprawling mountain view. Everything is balanced and peaceful. The children are touching the animals. A building with a tower stands nearby amidst abundant water and vegetation. The ample sky above is pregnant with anticipated divine promise.

As naturalistic as this scene might have been intended, it seems idyllic to us. Not that the milk cows, goats, and other animals would seem out of place in today’s herds or that divine promise is no longer needed. What is missing, unknown to the painter, is the zoonotic touch. Much has happened since the painter was revolutionizing the presentation of food-producing animals.

We have learned a few things, and not only about what cut short the painter’s life. Bovine tuberculosis, a long-time source of human infection, has declined in many parts of the world through control of the disease in cattle, affirming the application of “one medicine” for animal and human health. But progress in some is offset by new developments in other animal diseases, such as in the identification of a new orthobunyavirus in cattle in Europe and the newly defined European pathogen sheep in a devastating disease of New World bighorn sheep. In addition, chronic wasting disease continues to spread geographically among farmed and wild deer and elk in the United States and Canada, fanning concerns about its unknown zoonotic potential.

Many other infections continue to plague animals harvested as food and the humans who rely on them. For example, neurocysticercosis, a well-known neurologic disease in developing countries, is increasingly found in refugees in the United States, where the infection is not endemic. Recent outbreaks in Europe and the United States suggest an expanding role of bovine non-O157 *E. coli* strains in human disease. Unpasteurized dairy products continue to be associated with disease outbreaks in the United States. And for reservoirs and interspecies transmission of highly pathogenic viruses like Nipah virus and Hendra virus, the relationships between bats, humans, other animals, and the environment remain difficult to understand and control.

Despite increasing knowledge of the closeness of human and animal destiny, the “mutual obligation” so clearly defined by Charron is slow to advance. And, despite public health efforts, long follies persist for lack of adequate study or application of existing epidemiologic and biomedical tools.

**Figure:**
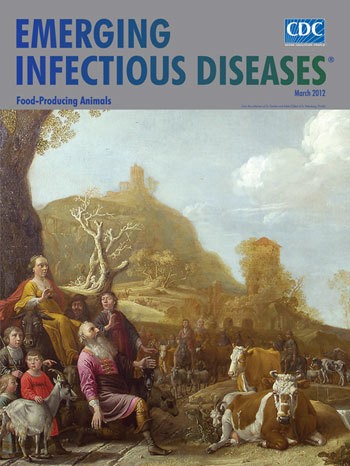
**Paulus Potter (c. 1625–1654) *God Appearing to Abraham at Sichem* (1642) (detail) Oil on canvas (100.4 cm × 130.8 cm)** From the collection of Dr. Gordon Gilbert, St. Petersburg, Florida, USA. Photo by Ray Bassett
